# Artificial Intelligence and Augmented Reality in Orthopedic Surgery: A Narrative Review of Current Applications and Future Directions

**DOI:** 10.7759/cureus.100177

**Published:** 2025-12-27

**Authors:** Hiram E Luigi-Martínez, Josué G Layuno-Matos, Naomy A Fernández-Vélez, Rafael Fernández-Soltero, Rafael Señeriz-Ortiz

**Affiliations:** 1 Department of Orthopaedic Surgery, Ponce Health Sciences University, Ponce, PRI

**Keywords:** artificial inteligence, augmented reality surgical navigation, computer vision, deep learning, machine learning, mixed reality, narrative review, natural language processing, neural network, ortho surgery

## Abstract

Artificial intelligence (AI) and augmented reality (AR), including mixed reality systems, are increasingly integrated across the orthopedic surgical continuum to enhance imaging interpretation, preoperative planning, intraoperative navigation, postoperative assessment, and surgical education. AI techniques such as machine learning, deep learning, and computer vision now facilitate automated segmentation, three-dimensional reconstruction, radiographic measurement, implant identification, and risk prediction with high accuracy across multiple subspecialties. AR platforms provide real-time visualization of anatomy, component alignment, and instrument trajectories, with studies in arthroplasty and spine surgery demonstrating placement accuracy within 1-2 degrees or a few millimeters, along with reduced fluoroscopy usage in selected procedures. Applications in trauma, oncology, pediatrics, and sports medicine similarly show feasibility for improving visualization, optimizing implant planning, and supporting complex resections. Educational uses of AR and mixed reality consistently improve technical performance and shorten learning curves among trainees. Despite these advances, most published studies involve small single-center cohorts, heterogeneous outcome measures, and limited external validation of AI models. AR systems also face practical challenges related to ergonomics, workflow integration, registration accuracy, and device usability. Overall, current evidence indicates that AI and AR have progressed from experimental concepts to early clinical utility across several orthopedic domains; however, broader adoption will require multicenter studies powered for patient-centered and economic outcomes, standardized reporting and validation of AI models, rigorous human-factors evaluation of AR interfaces, and development of interoperable platforms that integrate real-time AI analytics into ergonomic AR workflows. These efforts are essential to ensure the safe, effective, and equitable implementation of AI- and AR-enhanced orthopedic care.

## Introduction and background

Orthopedic surgery depends on a precise three-dimensional understanding of complex anatomy and the reliable execution of technically demanding procedures in constrained operative environments. Small deviations in component alignment, bone resection planes, or screw trajectory can alter joint biomechanics, accelerate wear, or jeopardize stability. At the same time, the increasing volume and complexity of orthopedic care demand efficient workflows and rational use of resources.

AI and AR offer complementary approaches to these challenges. AI encompasses a spectrum of computational techniques, including classical machine learning, deep learning with convolutional and other neural networks, computer vision, natural language processing (NLP), and reinforcement learning. In orthopedics, these methods support automatic image analysis, implant identification, segmentation, and three-dimensional reconstruction, preoperative planning, risk prediction, and analysis of electronic health record data [1,2,3,4,5,6,7,8,9,10]. AR refers to systems that overlay computer-generated information onto the user’s view of the real world, while MR blends physical and digital content in an interactive three-dimensional environment. Orthopedic applications typically use optical see-through head-mounted displays or video-based systems to project anatomical models, planned implant positions, or alignment axes directly onto the operative field or patient, synchronized with tracking systems [11,12,13,14,15,16,17,18].

In arthroplasty, AR navigation systems have been developed for total knee arthroplasty (TKA), total hip arthroplasty (THA), and shoulder arthroplasty to guide bone cuts, implant placement, and limb alignment [19,20,21,22,23,24]. In spine, AR has been applied for screw placement, deformity correction, and remote tele mentoring [15,17,18,25,26,27]. AI and AR are also explored in complex pelvic and acetabular fracture fixation, musculoskeletal oncology, and pediatric applications [9,16,25,28,29,30,31,32,33,34,35,36]. Beyond intraoperative guidance, AI-driven models support patient-specific instrumentation, three-dimensional planning, and postoperative surveillance in arthroplasty [2,7,10,35,37,38,39,40,41]. Machine learning algorithms predict complications, length of stay, and resource utilization to inform perioperative pathways and value-based care [5,6,37,38,42,43,44,45,46,47,48]. NLP tools identify adverse events, structure operative notes, and extract registry quality data from clinical documentation [48,49,50,51,52].

Despite these advances, several questions remain. First, to what extent do current AR and AI systems improve technical accuracy, safety, and patient-centered outcomes relative to established standards such as conventional navigation or robotics? Second, how mature and generalizable are AI models when applied outside of development institutions? Third, what practical, ergonomic, and regulatory barriers must be addressed to integrate these technologies at scale?

This narrative review addresses these questions by synthesizing current evidence on AI and AR in orthopedic surgery, highlighting applications with early clinical impact, identifying limitations of the existing literature, and outlining priorities for future research and implementation.

## Review

​​​​Methodology

We conducted a narrative review with structured elements, tailored to a broad and heterogeneous body of literature spanning multiple orthopedic subspecialties, technology classes, and outcome domains. To capture the full scope of relevant evidence, we included studies evaluating educational and simulation applications of AR and VR that enhance surgical performance, particularly for inexperienced surgeons [53]. This approach also allows integration of high-level evidence, including systematic reviews and randomized trials, alongside illustrative case series, technical feasibility studies, and horizon scanning work on emerging technologies.

Literature identification

The review was informed by two prior structured evidence syntheses that used semantic search to screen the orthopedic literature on: (1) combined AI and AR systems and their effect on patient care outcomes, and (2) the evolution of AI applications in orthopedic surgery from 2010 through 2023. These scans identified several hundred candidate publications, from which the highest-scoring studies were extracted and summarized, focusing on clinical applications and key technical themes.

Building on that foundation, we performed targeted searches of PubMed to identify additional original studies and reviews through May 2025. Search terms combined keywords and Medical Subject Headings related to AI and AR with orthopedic surgery, including: “artificial intelligence,” “machine learning,” “deep learning,” “neural network,” “computer vision,” “natural language processing,” “augmented reality,” “mixed reality,” “virtual reality,” “digital twin,” “orthopaedic,” “arthroplasty,” “spine,” “fracture,” “oncology,” “pediatric orthopaedics,” and “sports medicine.”

Reference lists of recent systematic and scoping reviews were screened to identify further primary studies in domains such as arthroplasty, spine, trauma, oncology, and education [2,4,5,7,8,9,12,13,14,18,21,37,48,50,54,55,56].

Eligibility criteria

We included clinical studies involving human patients undergoing orthopedic procedures where AI or AR contributed to preoperative planning, intraoperative navigation or guidance, postoperative surveillance, or rehabilitation; preclinical or technical studies using cadaveric models, phantoms, or validated simulations when they addressed navigation, alignment, or planning tasks relevant to orthopedic surgery; systematic reviews, scoping reviews, meta-analyses, and narrative reviews focused on AI or AR in orthopedics or related subspecialties; and publications appearing in peer-reviewed journals indexed in PubMed. We excluded studies focused solely on basic algorithm development without an orthopedic context, simulation studies unrelated to orthopedic procedures or anatomy, and editorials, commentaries, or letters lacking original data or substantive methodological detail.

Data extraction and synthesis

For each included study or review, we noted the orthopedic domain, technology type (AI, AR, MR, VR, robotics, NLP, digital twin), study design, sample size where applicable, clinical setting, primary outcomes, and key findings. AI applications were categorized by perioperative phase (preoperative imaging and planning, intraoperative guidance, postoperative surveillance and prediction, documentation and registry support, education and simulation), and AR applications by subspecialty and task (arthroplasty, spine, trauma, oncology, pediatric, training, teleassistance).

Given the diversity of designs and outcomes, no formal quantitative meta-analysis or risk of bias scoring was attempted. Instead, we provide a structured qualitative synthesis, emphasizing areas with higher-level evidence (randomized trials, prospective cohorts, meta-analyses) and clearly distinguishing them from early feasibility work.

Overview of AI and AR technologies relevant to orthopedics

AI Modalities

AI in orthopedics primarily uses supervised machine learning and deep learning.

Classical machine learning models (e.g., logistic regression with regularization, random forests, support vector machines, gradient boosting) have been applied to predict implant size, complications, readmission, and resource utilization [1,2,5,6,38,44,45,47,48]. Deep learning and convolutional neural networks dominate imaging applications, including fracture detection, osteoarthritis grading, implant identification, segmentation of bone and implants, and reconstruction of three-dimensional bone models from two-dimensional images. Digital twins and advanced modeling frameworks have also been explored as complementary tools, enabling simulation of joint mechanics and virtual surgery [5,7,8,10,41,46,57,58,59].

Computer vision methods enable automatic extraction of alignment parameters and radiographic measurements from standard radiographs, for example, mechanical axis, component orientation, or joint line height [7,10,46,59]. NLP systems extract structured information from operative reports and clinical documentation, detect adverse events, and facilitate registry-level data collection [48,49,50].

Digital twins and advanced modeling frameworks combine patient-specific imaging, kinematics, and sometimes sensor data to simulate joint mechanics and virtual surgery, with early applications in ankle and knee arthroplasty and broader orthopedic evaluation [5,57]. In arthroplasty, AI models have been embedded in commercial planning platforms that generate three-dimensional reconstructions, propose component sizes and positions, and interface with patient-specific guides or robotic systems [2,10,35,39,40,41,52].

Augmented and Mixed Reality Platforms

AR systems used in orthopedic surgery can be grouped into:

Optical see-through head-mounted displays that superimpose holographic content onto the surgeon’s direct view of the patient. Devices such as Microsoft HoloLens have been adapted for arthroplasty, spine screw placement, pelvic fixation, and oncologic resection [12,13,15,16,17,18,21,24,25,32,34,36,60]. Monitor or projector-based systems that overlay virtual trajectories or templates onto fluoroscopic or camera images without direct head-mounted visualization [14,18,23,36].

Mixed reality platforms that combine AR with immersive VR and bidirectional interaction, often integrating telepresence, multimodal imaging, and haptic or robotic components [13,16,17,18,32,60,61]. These systems rely on varying combinations of optical trackers, inertial sensors, surface mapping, and registration based on preoperative CT or intraoperative fluoroscopy. Key technical challenges include maintaining accurate registration despite soft tissue deformation, preserving sterility, ergonomics, and comfort of headsets, occlusion of the operative field, and latency or drift that can influence trust and usability [12,13,15,16,18,21,32,62,63].

Integration of AI with AR

Several groups have begun to embed AI-driven segmentation, registration, or decision support within AR workflows. Examples include vertebroplasty navigation combining AI-based targeting with AR overlays, MR-based screw placement with AI-enhanced visualization, and hybrid systems that use AI algorithms to convert two-dimensional images into three-dimensional models that are then projected intraoperatively [3,5,13,17,29,32,62,64]. Digital twins offer another integration pathway, where AI-derived models of joint mechanics inform AR overlays during planning and execution [5,57,62].

Clinical applications of AR and mixed reality: total joint arthroplasty

Total Knee Arthroplasty

AR navigation systems for TKA typically use a head-mounted display with optical tracking markers or surface mapping to provide real-time feedback on coronal and sagittal alignment, resection levels, and component positioning. A systematic review by Iacono et al. identified early clinical and preclinical studies of AR-assisted TKA and reported that contemporary systems achieved mechanical axis alignment and component positioning errors on the order of 1 to 2 degrees in most series [24]. A clinical pilot component of that review suggested feasibility and acceptable early outcomes but highlighted setup time and learning curve as important considerations.

Rossi et al. reviewed AR applications in total joint arthroplasty and found that in TKA, AR guidance improved cut accuracy relative to conventional instrumentation, with a proportion of cases achieving alignment within 3 degrees of neutral in the coronal plane comparable to or better than historical robotic or optical navigation cohorts [21,65,66]. Although sample sizes were modest and often single-center, these findings have been corroborated by subsequent series.

In a single-center prospective study of 76 consecutive TKAs, Castellarin et al. reported that an AR system improved femoral and tibial cut accuracy compared with their historical experience, without a significant increase in operative time once the initial learning curve had passed [20]. Minor technical and registration issues were noted early in adoption but decreased with familiarity. Similarly, Sakellariou et al. described clinical experience with an AR-based TKA navigation platform, reporting high accuracy of component placement and limb alignment with acceptable complication rates [19]. Their study highlighted the importance of intraoperative verification and calibration to mitigate cumulative drift in alignment measurements.

Collectively, these early studies suggest that AR navigation for TKA can deliver cut and alignment accuracy like that of conventional computer-assisted navigation and some robotic systems, with potentially lower hardware costs and more flexible deployment. However, most reports are single-center series or pilot studies, and robust randomized trials powered for patient-reported outcomes, revision, and cost effectiveness remain lacking [2,19,20,21,24].

Total Hip Arthroplasty

AR systems have also been developed for acetabular cup placement in THA. A retrospective comparative study evaluated an AR navigation system versus an accelerometer-based portable navigation device for acetabular cup placement during THA in the lateral decubitus position [22]. The AR group demonstrated non-inferior or improved accuracy of inclination and anteversion relative to target values, with similar complication rates.

Zubkov and Torchynskyi described the integration of MR technologies into hip arthroplasty, reporting accurate positioning of components and favorable short-term outcomes, while emphasizing that precise registration and ergonomic optimization of the headset were crucial for safe adoption [60]. Moreover, Rossi et al., in their review of THA case series, concluded that AR-assisted THA cup placement can achieve accuracy comparable to CT-based navigation, but the evidence base remains small and heterogeneous in terms of patient position, surgical approach, and measurement methods [16,18,21,22].

Shoulder Arthroplasty and Other Joints

In total shoulder arthroplasty, Longo et al. systematically reviewed applications of VR, AR, and the metaverse, identifying early experiences with AR-guided glenoid component placement and immersive VR training environments [55]. While technical feasibility and improved understanding of three-dimensional glenoid anatomy were reported, there is limited evidence that AR guidance improves clinical outcomes compared with existing navigation or patient-specific instrumentation. Additional reports have explored AR in total elbow arthroplasty and reverse shoulder arthroplasty, primarily as feasibility studies or small series summarized in broader AR reviews [12,18,21,55,56].

Spine Surgery and Vertebroplasty

AR has gained particular attention in spine surgery, where accurate trajectory and depth of pedicle screws, safe placement of vertebral augmentation devices, and complex deformity correction benefit from enhanced three-dimensional visualization. Cofano et al. reported early experience with AR in spine surgery and remote assistance. Using head-mounted displays, surgeons accessed holographic representations of imaging data and remote expert annotations during spinal procedures. The authors described improved anatomical understanding and workflow integration, but emphasized the importance of ergonomics, line of sight, and user training for successful implementation [15,26,28].

De Jesus Encarnacion Ramirez et al. reviewed AR integration in spine surgery, highlighting applications in screw placement, decompression, deformity correction, and tele-mentoring [32]. Across the included reports, AR systems generally delivered high accuracy of screw placement relative to conventional fluoroscopy or navigation, with reductions in radiation exposure in some series. However, most data were observational and lacked long-term follow-up. Furthermore, Auloge et al. conducted a pilot randomized trial of AR and AI-based navigation for percutaneous vertebroplasty versus standard fluoroscopic guidance [64]. The AR-assisted group achieved accurate needle placement with fewer fluoroscopy shots and lower radiation exposure, although setup time for the AR system partially offset intraoperative efficiency gains. No major differences in short-term complications were observed, but the study was underpowered for rare events.

Mixed reality meta-analyses and systematic reviews of AR in musculoskeletal and spine interventions corroborate these findings, reporting improved or non-inferior targeting accuracy, potential reductions in radiation exposure, and heterogeneous effects on operative time [13,14,16,17,18,32].

Trauma and Orthopedic Oncology

Complex trauma and oncologic reconstructions pose substantial challenges for conventional navigation. AR offers a way to visualize resection margins, planned osteotomies, and custom implants directly on the patient. Shen et al. described an AR system for patient-specific reconstruction plate design in pelvic and acetabular fracture surgery, demonstrating accurate plate adaptation and enhanced visualization of complex anatomy [25]. Although primarily a technical and preclinical series, the study illustrated the feasibility of integrating computer graphics-based models with intraoperative overlays.

Gupta and Ambade reviewed VR and AR in orthopedic trauma surgery, including applications in fracture reduction, screw placement, and preoperative rehearsal [54]. They concluded that AR and VR can support more precise implant positioning and improved understanding of fracture patterns, but emphasized that many studies are limited to simulation or small case series. In musculoskeletal oncology, Nasir et al. systematically reviewed AR for orthopedic and maxillofacial oncologic surgery, focusing on both clinical and technical aspects [34]. AR-based systems improved visualization of tumor margins and assisted in the placement of resection planes and reconstruction hardware, with reported reductions in resection error relative to preoperative plans. Wong et al. similarly summarized MR applications in orthopedic oncology, including limb salvage procedures and complex pelvic resections, and underscored the promise of MR for real-time navigation in anatomically complex regions [16].

Building on these findings, Lu et al. reported on a pilot series using MR technology in orthopedic surgery that combined head-mounted displays, fifth-generation (5G) connectivity, and robotic platforms to enhance intraoperative communication, screw placement, and patient education [17]. Their experience highlighted the potential of hybrid systems that merge MR, robotics, and telepresence in high complexity trauma and oncologic cases.

Education, simulation, and telepresence

A substantial portion of the AR and MR literature concerns education and simulation rather than direct intraoperative navigation. Demeco et al. conducted a meta-analysis of imaging-derived holograms used as adjuncts in surgical training and reported that such tools improved the performance of inexperienced surgeons, particularly in complex anatomic regions [53]. Although their work included non-orthopedic procedures, the principles apply to orthopedic training.

Goh et al. reviewed VR and AR-based surgical training and simulation in TKA, finding that immersive and AR-enhanced simulators improved objective performance metrics, accelerated skill acquisition, and supported better retention of spatial knowledge compared with traditional teaching or standard video-based instruction [67]. In addition, Longo et al. provided a broad systematic review of AR, VR, and AI in orthopedic surgery, including training applications [56]. They concluded that extended reality technologies could shorten learning curves across several procedures, particularly for residents and fellows, but noted that translation of improved simulation performance to patient outcomes remained largely untested.

Stevanie et al. evaluated MR in orthognathic surgery and described improvements in accuracy, operative time, and skill acquisition in training environments, alongside cost and technical constraints [61]. Although focused on maxillofacial surgery, the lessons regarding MR implementation and valuation of training benefits apply to orthopedics. Furthermore, Vavra et al. reviewed AR in surgery more broadly and emphasized its role in education and tele-mentoring, summarizing early examples of remote guidance through shared AR visualizations [18]. Likewise, Cofano et al. and De Jesus Encarnacion Ramirez et al. described mixed reality teleassistance in spine surgery, with remote experts annotating holographic views of the operative field to support on-site surgeons, illustrating the potential for democratizing access to highly specialized expertise [15,32].

Overall, educational and telepresence applications of AR and MR show consistent improvements in trainee performance and surgeon satisfaction, but the evidence is dominated by simulation studies and early clinical experience.

AI applications across the orthopedic perioperative pathway

Preoperative Imaging, Segmentation, and Three-Dimensional Planning

AI has transformed preoperative imaging workflows in arthroplasty by automating segmentation, three-dimensional reconstruction, and templating. Lambrechts et al. developed an AI-based patient-specific planning algorithm for TKA that uses machine learning regression models to predict optimal component size and position [**36**]. Their system improved agreement between planned and implanted component sizes and reduced the need for intraoperative adjustments relative to conventional templating.

Fernandes et al. evaluated an AI algorithm that converts standard two-dimensional radiographs to three-dimensional bone models for TKA [10]. They reported high accuracy, reliability, and repeatability of reconstructed models relative to CT-based ground truth, supporting the potential to reduce preoperative CT requirements and radiation exposure. Alongside this, Marsilio et al. introduced a combined edge loss U-Net architecture for optimized knee segmentation in preoperative TKA planning, demonstrating high Dice coefficients and robust performance across varying image quality [41].

In THA, Chen et al. described the development and validation of an AI-based preoperative planning system that segments pelvic and femoral anatomy, simulates implant placement, and provides patient-specific recommendations [40]. Prospective evaluation showed accurate prediction of component sizes and satisfactory radiographic outcomes. Zhang et al. evaluated three-dimensional AI-based preoperative planning for primary THA in a retrospective cohort and reported that both junior and experienced surgeons benefited from improved accuracy of implant positioning and reduced planning time [39].

Rouzrokh et al. developed THA Net, a deep learning solution that supports next-generation templating and patient-specific surgical execution in THA, further illustrating the trend toward fully integrated AI planning pipelines [52]. Systematic reviews and scoping reviews have confirmed the rapid adoption of AI tools for segmentation, templating, and three-dimensional planning in arthroplasty, reporting strong performance metrics in terms of segmentation accuracy and planning reliability, although external validation remains variable. Additional studies have also explored AI applications in fracture detection and musculoskeletal imaging [2,7,10,41,46,50].

Postoperative Imaging, Implant Identification, and Alignment Analysis

AI also automates postoperative radiographic assessment and implant surveillance. Shah et al. systematically reviewed AI applications for implant analysis in total joint arthroplasty and found that convolutional neural networks could accurately classify implant type, manufacturer, and sometimes specific models from standard radiographs, often with accuracy exceeding 90% [68]. Ren and Yi similarly reviewed AI for orthopedic implant model classification and reported that deep learning-based systems achieved near-expert-level performance in identifying a variety of implant designs [58].

A scoping review of AI-based image analysis in THA and TKA demonstrated applications, including implant identification, wear assessment, and alignment measurement, concluding that these tools may reduce manual workload and support large-scale registry-based research [8]. Bernard de Villeneuve et al. trained a convolutional neural network to analyze lower limb alignment on radiographs and found that their AI-based approach provided precise and reproducible measurements that could standardize deformity analysis [59]. Bonnin et al. evaluated an AI radiographic analysis tool for TKA and reported accurate detection of radiographic parameters and detection of outliers, supporting its use as a supplement to surgeon assessment [46].

Collectively, this body of work indicates that AI can reliably automate many repetitive radiographic tasks in arthroplasty and deformity analysis, which may facilitate quality assurance, registry data curation, and longitudinal surveillance.

Diagnostic Imaging and Fracture Detection

Diagnostic imaging is one of the most mature domains for AI in orthopedics.

A systematic review of artificial intelligence methods for fracture detection and classification in orthopedic trauma imaging reported very high-performance metrics across multiple studies, with areas under the curve ranging from 0.95 to 1.0 and accuracy frequently between 83% and 98% [7]. These models have been applied to wrist, ankle, hip, and proximal humerus fractures, among others.

Korneev et al. reviewed AI in preventive orthopedics and described models that predict the risk of osteoarthritis progression and the need for arthroplasty based on imaging and clinical features [5]. Prudnikov et al. summarized AI applications in the diagnosis and treatment of orthopedic diseases, noting that AI can assist in detecting degenerative changes, classifying rotator cuff tears and ligament injuries, and supporting treatment selection [27]. Lee et al. reviewed AI in the diagnosis of knee osteoarthritis and prediction of arthroplasty outcomes, concluding that AI-based models can outperform traditional radiographic grading in predicting progression and surgery, but that heterogeneity of datasets and limited external validation remain major limitations [47].

Risk Prediction, Outcome Modeling, and Resource Optimization

AI models for perioperative risk prediction and outcomes analysis have proliferated in arthroplasty and spine surgery. Hornung et al. reviewed AI applications in spine care and noted that machine learning models predict surgical complications, reoperations, and patient-reported outcomes based on demographic, radiographic, and operative variables, often outperforming conventional regression [6]. Lopez et al. reported similar findings in a systematic review of machine learning in spine surgery, although many models were developed in single-center datasets [42]. In spine surgery, systematic reviews have described AI tools capable of predicting surgical outcomes, deformity correction success, and complication risk, including applications in minimally invasive adult spinal deformity surgery [44,45]. These models support personalized surgical planning and shared decision-making, although external validation and integration into clinical workflows are still limited.

In arthroplasty, Entezari et al. systematically reviewed machine learning and optimization approaches to improve resource utilization and pathway design in arthroplasty care, including prediction of length of stay, discharge disposition, and readmission [38]. Polce et al. reviewed AI and machine learning analyses in TJA and concluded that predictive models hold promise for risk stratification and personalized care but often suffer from limited generalizability and a lack of transparent reporting [48].

Korneev et al. discussed preventive orthopedic applications, including prediction of osteoarthritis onset and progression, while Polisetty et al. focused on concerns and recommendations for meaningful AI adoption in hip and knee arthroplasty [5,51]. These reviews highlight both the potential and the need for robust validation, fairness assessment, and careful interpretation of AI predictions.

Subspecialty applications: sports, shoulder, foot and ankle, pediatric, oncology

AI is increasingly applied across orthopedic subspecialties beyond arthroplasty and spine. In sports medicine, Andriollo et al. reviewed AI in anterior cruciate ligament injuries, summarizing models for injury prediction, diagnosis, graft choice, and rehabilitation monitoring [31]. Correlated work in rotator cuff disease, reviewed by Velasquez Garcia et al., describes models for tear detection, classification, and prognosis that may support more individualized treatment [69]. Gupta et al. produced systematic reviews of AI applications in shoulder surgery and foot and ankle surgery. These works identified AI models for outcome prediction after rotator cuff repair, shoulder arthroplasty, ankle and hindfoot procedures, and fracture fixation, but concluded that the evidence base remains early, with relatively small cohorts and rare external validation [70,71].

Rumaih et al. reviewed AI in pediatric orthopedic surgery and reported applications in scoliosis detection, limb deformity analysis, fracture classification, and risk prediction. They emphasized that issues of data sparsity, growth-related anatomical changes, and fairness in pediatric datasets require particular attention [33].

In musculoskeletal oncology, Li et al. systematically reviewed AI in musculoskeletal oncology and found that machine learning models can aid in tumor detection, histologic classification, response prediction, and survival estimation, while AR and MR support intraoperative visualization and margin control [9,16,34].

Documentation, NLP, and registry support

NLP and related AI tools are increasingly used to structure orthopedic documentation and support registries. Sasanelli et al. conducted a scoping review of NLP tools in orthopedic surgery and identified systems that extract diagnoses, procedures, complications, and outcome measures from clinical text with high F1 scores and sensitivities [49]. Applications included adverse event detection, automated registry submission, and quality reporting. Moreover, Federer and Jones, as well as Polce et al., highlighted the role of AI in summarizing and structuring large volumes of clinical data, but warned that robust governance, transparent model development, and close clinician oversight are required to avoid misclassification and bias [48,50].

Combined AI and AR systems

The integration of AI and AR into unified platforms represents an important frontier. Auloge et al.’s pilot randomized trial in percutaneous vertebroplasty is a representative example, where AI and AR are combined in a navigation system that uses AI algorithms to assist trajectory planning and AR overlays to guide needle insertion [64]. The study demonstrated accurate targeting and reductions in fluoroscopy usage relative to standard care, but highlighted setup time, calibration requirements, and the need for specialized training. Similarly, Lu et al. presented a mixed reality pilot study that combined MR headsets, AI-enhanced imaging, fifth-generation connectivity, and robotics in orthopedic surgery [17]. They reported improved screw placement accuracy, enhanced communication among team members, and high surgeon satisfaction.

Beyond these early clinical examples, Oettl et al. discussed the broader concept of multimodal AI models and continuous monitoring in orthopedics, envisioning systems that integrate imaging, wearable sensors, and clinical data with AR-based visualization to support intraoperative and postoperative decision-making [62]. Dean et al. described digital twins for orthopedic evaluation and treatment, which could serve as substrates for AI-driven simulation and AR visualization, enabling rehearsal and intraoperative adaptation of patient-specific plans [57]. Misir and Yuce, Kumar et al., and Baghbani et al. also discussed digital twins and combined AI AR robotics ecosystems as promising directions for future orthopedic care [1,3,4].

Although these integrative approaches remain largely in early clinical or preclinical stages, they exemplify how AI and AR can be co-deployed rather than used in isolation, highlighting a pathway toward more precise orthopedic interventions.

Practical implementation considerations

Workflow Integration and Ergonomics

Successful adoption of AI and AR tools depends on seamless integration into existing workflows. Chiu et al. described the challenges of implementing AR in a busy orthopedic department, including the need for training, workflow redesign, and addressing device ergonomics and user discomfort [63]. Bollen et al., Rossi et al., and **Rito **et al. observed that AR and MR systems can increase setup time and sometimes prolong operative duration during early adoption, although time differences often diminish with experience [13,14,21]. Headset weight, field of view, occlusion of instruments or staff, and difficulty maintaining a sterile field around wearable devices are recurrent themes [12,13,15,16,18,21,32,63].

AI systems for planning and prediction must be tightly integrated with hospital imaging archives, electronic health records, and existing navigation or robotic platforms. Reviews by Batailler et al., Korneev et al., and Polce et al. emphasize the importance of user-friendly interfaces, clear presentation of model outputs, and avoidance of alert fatigue [2,5,48].

Data Quality, Bias, and Validation

Many AI models are trained on retrospective data from single institutions, raising concerns about overfitting and bias. Federer and Jones, Korneev et al., Polce et al., and Polisetty et al. stress that robust external validation, calibration, and transparent reporting are essential before clinical deployment [5,48,50,51].

Datasets used for training may underrepresent specific demographic groups, rare conditions, or pediatric populations, potentially leading to inequitable performance. Reviews focusing on pediatric orthopedics, as well as shoulder, foot, and ankle surgery, emphasize the importance of careful dataset curation and subgroup analyses to ensure reliable performance of artificial intelligence models across diverse patient populations [33,70,71].

Regulation, Ethics, and Medico-legal Considerations

The regulatory landscape for AI and AR is evolving. Reviews by Misir and Yuce, Baghbani et al., Kumar et al., and others highlight issues such as classification of AI tools as medical devices, the need for post market surveillance of adaptive algorithms, data protection, and responsibility when AI recommendations conflict with clinician judgment [1,3,4,5,50,51].

AR systems introduce additional concerns regarding distraction, cognitive load, and potential overreliance on virtual overlays. Ensuring that surgeons maintain situational awareness and retain the ability to operate safely in the event of system failure is critical.

Future directions and research priorities

Several themes emerge from the current literature that can guide future work.

From Technical Accuracy to Patient-Centered Outcomes

Most AR and AI studies emphasize alignment, targeting accuracy, or model performance metrics. Few are powered to detect differences in revision, complications, pain, function, or quality of life. Multi-center randomized or pragmatic trials that compare AI and AR augmented workflows against best available standard care and evaluate long-term patient-reported outcomes and cost effectiveness are needed [2,5,6,7,13,14,19,20,21,37,48,51,55,56].

Standardized Reporting and Validation of AI Models

Researchers should adopt standardized reporting frameworks for AI in medicine, provide transparent descriptions of datasets and model architectures, and perform external validation in distinct populations. Calibration, fairness assessment, and clear communication of uncertainty are crucial for safe deployment [5,6,42,44,48,50,51].

Robust Evaluation of AR and MR Ergonomics and Human Factors

Beyond measuring alignment accuracy, studies should systematically assess surgeon workload, eye strain, physical comfort, usability, and learning curves associated with head-mounted displays and AR interfaces. Human factors research can guide industrial design and optimize the balance between information richness and cognitive load [12,13,15,16,18,21,32,63].

Combination of AI, AR, Robotics, and Digital Twins

Integrative platforms that couple AI-based planning and prediction with AR guidance, robotic execution, and digital twin simulation are likely to define future orthopedic workflows [3,4,13,17,57,62]. Rigorous evaluation of these ecosystems should consider not only accuracy but also interoperability, failure modes, and resilience.

Education, Credentialing, and Equitable Access

AR and VR simulation combined with AI-driven performance analytics may support competency-based training and credentialing, particularly in complex procedures [17,18,53,55,56,61,67]. Ensuring that trainees across institutions and regions have access to such tools is important to avoid widening disparities in surgical education.

Ethical and Societal Implications

Widespread deployment of AI and AR will influence the roles of surgeons, trainees, and allied personnel, and may alter patient expectations. Stakeholders must address consent for AI-assisted care, transparency about the role of algorithms, and appropriate sharing of value generated by data-driven systems (Tables 1-2) [1,3,4,5,50,51].

**Table 1:** Representative clinical and perioperative applications of augmented and mixed reality in orthopedic surgery. AR, augmented reality; MR, mixed reality; VR, virtual reality; AI, artificial intelligence; TKA, total knee arthroplasty; THA, total hip arthroplasty

Indication or context	Technology and platform	Study design and setting	Primary outcomes	Key findings	Representative references
Total knee arthroplasty alignment and cuts	Head-mounted AR TKA navigation systems	Systematic review with clinical pilot studies, single-center prospective cohorts	Mechanical axis alignment, component orientation, resection accuracy, complications	AR systems achieve sub-degree to low-degree errors in component alignment and resection accuracy with acceptable complication rates and learning curves.	[19,20,21,23,24]
Total hip arthroplasty acetabular cup placement	AR navigation, optical see-through, and MR systems, lateral decubitus THA	Retrospective comparative study, case series, systematic reviews	Cup inclination and anteversion, outliers relative to target safe zones, complications	AR navigation is non-inferior or superior to accelerometer-based portable navigation with similar complication rates; approaches demonstrate feasibility and accurate placement	[16,18,21,22,60]
Shoulder arthroplasty	AR and VR training modules, early AR glenoid guidance	Systematic review of extended reality in total shoulder arthroplasty, case reports	Accuracy of glenoid component positioning, training outcomes, complication	Extended reality improves understanding of glenoid anatomy and training performance; evidence for clinical benefit of intraoperative AR remains limited	[18,55,56]
Percutaneous vertebroplasty	Combined AI and AR navigation for vertebroplasty	Pilot randomized clinical trial	Targeting accuracy, fluoroscopy use, radiation dose, operative time, and complications	AR and AI guidance achieves accurate targeting with reduced fluoroscopy use; initial setup time offsets intraoperative efficiency gains.	[64]
Spine instrumentation and decompression	AR and MR guidance remote teleassistance	Case series, systematic reviews, narrative reviews	Screw placement accuracy, radiation exposure, workflow, usability, and complication	AR and MR systems support accurate screw placement with potential radiation reduction and enable remote assistance; ergonomics and learning curve are key	[13,14,15,17,18,32]
Pelvic and acetabular fracture fixation	AR patient-specific reconstructions, plate design, and trajectory guidance	Technical and preclinical series, early clinical reports	Fit reconstruction plates, screw trajectory accuracy, and complications	AR-enhanced plate design and visualization improve implant fit and trajectory planning; clinical validation remains limited	[18,25,29,54]
Musculoskeletal oncology resections	AR and MR for margin visualization and navigation	Systematic reviews and case series	Resection accuracy, margin status, complication	AR and MR improve visualization of tumor margins and reconstructive planning; early data suggest promising margin control.	[16,34]
Education and training across orthopaedics	AR and MR simulation, holographic models, VR environments	Randomized and controlled training studies, systematic reviews, and meta-analyses	Skill acquisition metrics, time to proficiency, knowledge retention, satisfaction	AR and MR training tools improve technical performance and shorten learning curves, especially for novice surgeons	[17,18,53,55,56,61,67]
Remote assistance and telepresence	MR-based teleassistance platforms in spine and general orthopaedics	Case series and feasibility reports	Workflow integration, feasibility, and user acceptance	MR systems enable remote experts to annotate the operative field and support on-site surgeons; acceptance depends on ergonomics and connectivity	[15,17,18,32]

**Table 2:** AI applications across the orthopaedic perioperative pathway. TKA, total knee arthroplasty; THA, total hip arthroplasty; AR, augmented reality; AI, artificial intelligence; CT, computed tomography

Perioperative phase	Task or domain	AI methods used	Representative performance and outcomes	Representative references
Preoperative imaging	Bone and implant segmentation, three-dimensional reconstruction for TKA and THA	Deep learning, U-Net variants, computer vision	High Dice coefficients for bone segmentation; accurate reconstruction of three-dimensional models from CT or radiographs	[41,47,60,62,63]
Preoperative planning	Templating, component sizing, patient-specific instrumentation, three-dimensional planning	Machine learning regression, deep learning, rule-based, plus learning systems	Improved prediction of component size and alignment; fewer intraoperative changes compared with conventional planning; reduced planning time	[2,10,35,39,40,41,52]
Intraoperative guidance	Trajectory planning, component alignment, integration with AR and robotics	Computer vision, AI-enhanced registration, multimodal AI	High targeting accuracy and alignment within 1 or 2 degrees in arthroplasty and spine; potential reductions in fluoroscopy when integrated with AR	[3,4,13,17,29,32,62,64]
Postoperative surveillance	Implant identification, radiographic parameter measurement, and alignment analysis	Convolutional neural networks, computer vision	High accuracy in implant classification; precise and reproducible measurement of alignment parameters; automated detection of outliers	[7,10,46,58,59,68]
Risk and outcome prediction	Prediction of complications, revision, functional outcomes, and resource utilization	Classical machine learning, neural networks, and ensemble models	Reported areas under the curve ranging from approximately 0.75 to 0.99 in several cohorts; improved prediction versus traditional regression in many studies	[5,6,38,42,44,45,47,48,51]
Diagnosis and triage	Fracture detection, osteoarthritis grading, ligament and tendon injury classification	Deep learning, convolutional neural networks	High diagnostic accuracy for fractures and degenerative diseases, sometimes matching or exceeding expert readers in controlled settings.	[5,8,27,47,69]
Subspecialty applications	Sports, pediatric, foot and ankle, shoulder, musculoskeletal oncology	Mixture of classical and deep learning methods	Models for injury diagnosis and prognosis, prediction of surgical outcomes, and survival estimation; evidence base is still early, with few external validations	[9,31,33,69,70,71]
Documentation and registry support	Event detection, note structuring, automated data extraction	Natural language processing, machine learning, deep learning	High F1 scores and sensitivity for identification of complications and key clinical variables; potential to streamline registry reporting	[48,48,50]
Continuous monitoring and digital twins	Longitudinal monitoring, virtual rehearsal, and simulation of joint mechanics	Multimodal AI, digital twin frameworks	Conceptual and early empirical work suggesting potential for personalized simulation and intraoperative adaptation	[3,4,5,57,62]

## Conclusions

AI and AR have evolved from early prototypes to tangible clinical tools in orthopedic surgery, particularly in arthroplasty and spine, with AR and MR navigation enabling precise component alignment and trajectory guidance, often reducing radiation exposure, and AI supporting imaging, preoperative planning, outcome prediction, and documentation across the perioperative pathway. Despite this progress, the evidence base is limited by small, often single-center studies, heterogeneous endpoints and platforms, sparse evaluation of clinical and economic outcomes, and many AI models lacking external validation. AR systems also face ongoing challenges with ergonomics, workflow integration, and cost. The next decade should emphasize rigorous clinical trials, standardized reporting and validation of AI tools, comprehensive evaluation of human factors for AR and MR, and development of interoperable platforms integrating real-time AI with intuitive AR guidance and, where appropriate, robotic execution. Thoughtful advancement in these areas has the potential to enhance technical precision, improve patient outcomes, and promote equity in orthopedic care.
